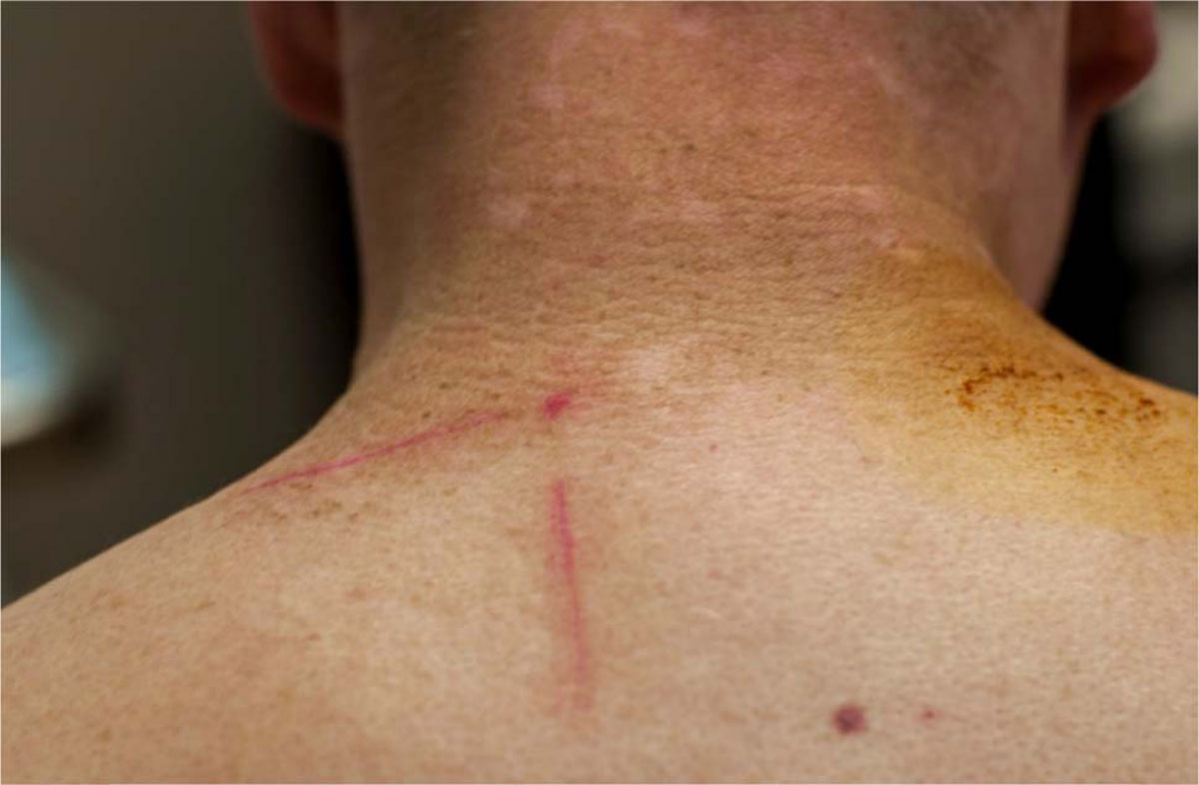# Complications of sequential immunotherapy in metastatic melanoma

**DOI:** 10.1186/2051-1426-3-S2-P128

**Published:** 2015-11-04

**Authors:** Shams Bufalino, Shruti Singh, Ewa Borys, Kelli Hutchens, I Caroline Le Poole, Joseph I Clark

**Affiliations:** 1 Loyola University Medical Center, Division of Hematology Oncology, Maywood, IL, USA; 2 Loyola University Medical Center, Division of Internal Medicine, Maywood, IL, USA; 3 Loyola University Medical Center, Department of Pathology, Maywood, IL, USA; 4 Loyola University of Chicago Health Sciences Division, Oncology Institute, Maywood, IL, USA

## Background

Advanced melanoma is associated with a poor prognosis. Targeted therapy and immunotherapy are mainstays of treatment. High-dose interleukin-2 (HD IL-2), interferon alfa-2b, cytotoxic T-lymphocyte-associated protein 4 (CTLA-4) inhibitors, and programmed cell death protein 1 (PD-1) inhibitors are approved immunotherapies. Genetically engineered T cells targeting melanoma antigens are currently under investigation. Patients with advanced melanoma are often exposed to multiple immunotherapies over their disease course. Adverse implications of sequential use of these therapies is not well described. Here we present a case of a patient with metastatic melanoma who developed rhabdomyolysis and vitiligo after various courses of immunotherapy.

## Methods

This is a 42 year old male with BRAF wild type melanoma metastatic to axilla, lung, and small bowel. Initial treatment consisted of four doses of ipilimumab with disease progression. He then enrolled in a clinical trial using fludarabine and cyclophosphamide conditioning for genetically engineered autologous T cells targeting tyrosinase. He experienced disease response lasting a few months but then progressed. After three doses of pembrolizumab, his disease again progressed, and he therefore initiated HD IL-2. During the first course of HD IL-2 he developed diffuse muscle pain due to rhabdomyolysis. HD IL-2 was discontinued with resolution in symptoms and lab abnormalities. Follow up imaging revealed a mixed response and he was again challenged with HD IL-2. He again rapidly developed rhabdomyolysis that resolved with discontinuation of therapy and hydration. A muscle biopsy was consistent with necrotizing myopathy with macrophage and T cell infiltration. Repeat imaging two months later revealed resolution of axillary lymphadenopathy and lung nodules that decreased in size. He was noted to have new onset diffuse vitiligo. A skin biopsy revealed diffuse CD3+ T cell infiltrate.

## Conclusions

The complications of sequential immunotherapy in metastatic melanoma has not been well described. Our patient experienced rhabdomyolysis after HD IL-2, which followed ipilimumab, genetically engineered T cells and pembrolizumab, respectively. It is unclear if previous immunotherapy, especially genetically engineered T cells, contributed to this unusual toxicity. Ultimately our patient experienced a favorable clinical response as evidenced by the development of vitiligo and decrease in his tumor burden. Vitiligo is an autoimmune process that may have been enhanced by his previous exposure to various immunotherapies. Physicians should be alert for rare and unexpected toxicities in patients who have received multiple lines of immunotherapy.

Written informed consent was obtained from the patient for publication of this abstract and any accompanying images. A copy of the writen consent is available for review.

**Figure 1:**
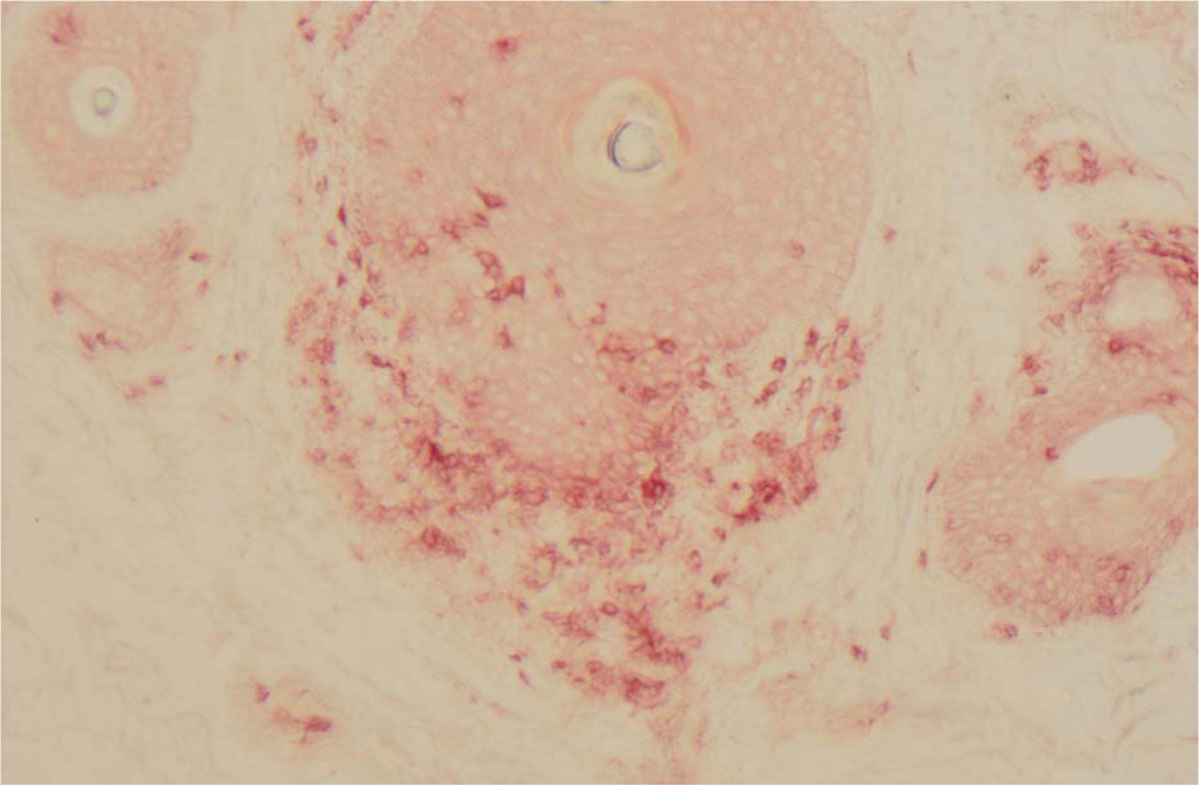


**Figure 2:**